# Investigating effects of day-to-day variations in environmental exposure on the human brain: study protocol for the Day2Day Environment project

**DOI:** 10.1186/s12868-026-01052-z

**Published:** 2026-09-23

**Authors:** Kim Falkenstein, Claire Pauley, Simone Kühn

**Affiliations:** 1 Center for Environmental Neuroscience, Max Planck Institute for Human Development, Lentzeallee 94, 14195 Berlin, Germany; 2 Faculty of Life Sciences, Humboldt-Universität zu Berlin, Invalidenstraße 42, 10115 Berlin, Germany; 3 Department of Psychiatry and Psychotherapy, University Medical Center Hamburg-Eppendorf, Martinistraße 52, 20251 Hamburg, Germany

**Keywords:** Environmental neuroscience, Variability, MRI, Brain structure, Brain function, Geographic ecological momentary assessment, Wearable sensors

## Abstract

**Background:**

Understanding how daily environmental exposures influence the human brain and mental health is increasingly important in the context of global urbanization and climate change. Previous studies have focused primarily on long-term environmental factors or isolated features like green space and air quality, neglecting the interaction between multiple exposures encountered in everyday life. This protocol paper describes the study design and dataset of the Day2Day Environment project, an ongoing study investigating how real-world, day-to-day environmental variability affects brain structure, function, and well-being.

**Methods:**

This dense longitudinal study collects multimodal data from 30 participants over 25 testing sessions, including neuroimaging and tracking of physiological, behavioral, and environmental variables. Using wearable devices, geographic ecological momentary assessments, and GPS-based geospatial tracking, we collect data on air quality, noise, light exposure, physical activity, stress, cognition, and affect. Neuroimaging measures include T1- and T2*-weighted images during rest and tasks, quantitative multi-parameter mapping, high-resolution proton density imaging of the hippocampus, and diffusion tensor imaging sequences.

**Conclusion:**

The Day2Day Environment dataset offers a unique opportunity to study short-term neuroplasticity in response to the dynamic interplay between multiple environmental exposures. Furthermore, the diversity of measurements in this dataset may be valuable for addressing research questions beyond environmental neuroscience.

## Background

With the rapid changes in our environment driven by global urbanization and climate change, it is crucial that we understand how different living and environmental characteristics impact mental and brain health. The emerging field of environmental neuroscience aims to identify and investigate the “active ingredients” of the physical environment — specific features, such as air quality, ambient noise levels, light exposure etc., that affect the working mechanisms of our brains and hence our mental well-being [1]. However, many studies focus on only one or a few isolated environmental factors, such as green space [e.g. 2–4] or air pollution [e.g. 5–7], which does not reflect the complexity of real-life exposure. Here, we introduce an ongoing study which comprises a comprehensive assessment of the physical environment in order to understand how these individual factors and their interactions impact brain structure and function.

### Long-term vs. short-term environmental effects on the brain

Several studies have investigated the effects of long-term living environments comparing the brain function and structure of individuals living either in cities, towns, or rural regions. One result of these studies indicated that individuals living in urban environments show higher brain activity in stress-related brain regions [8, 9], and structural deficits, namely reduced grey matter volume in the dorsolateral prefrontal cortex and pregenual anterior cingulate cortex associated with urban exposure during the first 15 years of life [10–12]. Existing research often simplifies the complexities of living environments into simpler categories such as “urban” and “rural” and focuses on isolated factors (e.g. air pollution, noise pollution, natural and artificial lighting etc.; [2, 13–15]). However, these factors are highly interrelated, with changes in one factor, such as green space availability, potentially influencing others like air quality or noise pollution. Therefore, it is important to consider both their common and unique contributions to brain structure and function.

In contrast to studies focusing on the effects of long-term living environments, short-term intervention studies (e.g. walks in nature) have demonstrated nature’s positive effect on enhancing working memory [16] as well as on stress, evident in physiological indicators and a reduction in activity in stress-related brain regions [17–19]. These findings underscore the importance of also studying short-term environmental effects on health and brain well-being. More significantly, it has been demonstrated that measures of brain structure and function are influenced by daily physiological changes in non-experimental situations, such as exercise [20], (de)hydration [21, 22], caffeine intake [23, 24], or the menstrual cycle [25]. These findings raise the question of whether day-to-day variations in physical environments could similarly impact brain structure and function.

### Reliability of measurement

In longitudinal neuroimaging research, reliably detecting small differences in brain structure is often constrained by measurement variability [26]. Even with consistent scanner settings, repeated magnetic resonance imaging (MRI) of the same individual within a short time often yield differences in neuroimaging parameters [27]. This short-term variability is often interpreted as noise; however, it may rather arise from participants’ physiological and psychological states or constantly changing environmental factors. If confounding factors vary systematically across time points or individuals, the observed differences in MRI images may reach statistical significance. However, these differences may represent only temporary fluctuations rather than true individual differences or enduring changes. For this reason, the present dataset might also be used to estimate the baseline variability of brain parameters to compare plastic changes in the context of intervention studies. Likewise, the present dataset might be useful to test the stability of parameters derived from MRI data using different MRI processing pipelines.

Within the scope of the Day2Day study focusing on the reliability of MRI, an extensive MRI dataset was acquired over the course of six months from a cohort of younger adults (*n* = 6, ~ 50 MRI scanning sessions per participant; [28]). This study yielded two novel findings. First, robust predictors of within-person variance in grey matter and total cortex volume were identified: time of day and days since the first scan. While days since the first scan may indicate age-related changes rather than measurement error, time of day may be influenced by factors like fluid redistribution, hydration levels, and environmental conditions such as the temperature inside the MR scanner, although the underlying mechanisms are still speculative [29]. Additionally, this dataset has also become a valuable resource for the broader scientific community to explore a wide range of research questions, resulting in 11 publications to date [28–38]. For example, one study showed a positive association between the time spent outdoors (within 24 h before the MRI measurement) and grey matter volume in the right dorsolateral prefrontal cortex [38]. Crucially, these findings strongly suggest that rapid structural changes in the brain may occur in response to varying environments and behaviors, underscoring the importance of how we interact with our physical environment on a daily level.

However, the Day2Day study included only a very limited environmental assessment, leaving open the question as to how exposure to various environments (e.g. urban vs. green space) induces short-term neural changes. In order to elucidate the specific environmental factors driving neuropsychological variability, we initiated the Day2Day Environment project.

### The Day2Day Environment project

In this protocol paper, we outline the methodology of the Day2Day Environment project, which builds upon the previous Day2Day study from the same lab, with a larger sample and a broader set of variables, incorporating additional brain measures alongside various environmental, physiological, and behavioral factors that are monitored over time. These factors include air quality, ambient noise levels, light exposure, spatial context (e.g., green versus urban spaces, indoor versus outdoor), physical activity, stress levels, and emotional states. In terms of brain measures, we collect T1- and T2*-weighted images during rest and tasks, quantitative multi-parameter mapping, high-resolution proton density (PD) imaging of the hippocampus, and diffusion tensor imaging (DTI) sequences. These diverse measures enable us to investigate day-to-day variations in environmental exposure and their potential effect on brain structure, function, affect, and cognition. Furthermore, this dataset aims to advance the fields of environmental neuroscience and neuroimaging by providing valuable data for investigating within-person variability and reliability.

## Construction and content

### Participants

We aim to recruit a sample of 30 participants for this study. The planned sample size was determined based on a simulation of standard errors for within-person analyses, using data from the previous Day2Day study [28], conducted in Onyx. Recruitment initially targeted individuals who live in close proximity to the Max Planck Institute for Human Development in Berlin or are closely connected to the institute, using word of mouth, posters, and flyers. As data collection progressed, we broadened the recruitment strategy to include participants recruited via our institute’s participant database, who are not otherwise connected to the institute. Regardless of the recruitment channel, the study design requires significant effort from the participants and commitment over several months.

### Procedure

Interested participants contact the research team. This is followed by an online meeting to inform them in detail about the study procedure, to assess their eligibility for participation, and to set clear expectations about what participation would involve, i.e., that participation requires a high level of commitment.

Inclusion criteria: Participants must be between 18 and 50 years old and willing to undergo 25 testing sessions, including MRI scanning in each session. They must also commit to reliably wearing sensors and to use apps tracking various environmental, physiological, and behavioral factors for 24 h before each MRI appointment (hereon referred to as the pre-scan period).

Exclusion criteria: To assess individuals’ mental health status and identify signs or symptoms of psychological disorders, we use the Mini-DIPS screening tool including an online questionnaire and a clinical interview to exclude potential participants with current psychiatric disorders [39, 40]. Other exclusion criteria include the presence of neurological disorders and current or past addiction to alcohol and/or drugs. MRI-related exclusion criteria encompass having a cardiac pacemaker, metal implants (such as bone plates, joint prostheses, magnetic anal sphincter, vessel clips, artificial heart valves, stents, or cava filters), shards of metal from accidents or war injuries, fixed braces, tattoos above the shoulders, permanent make-up, intrauterine devices for contraception which contain metal, hearing prostheses, medication or drug pumps, acupuncture needles, and other metallic foreign bodies that cannot be removed. Additionally, pregnancy or possible pregnancy, previous brain and/or heart surgery and experiencing difficulty in confined spaces are criteria for exclusion.

**Fig. 1:**
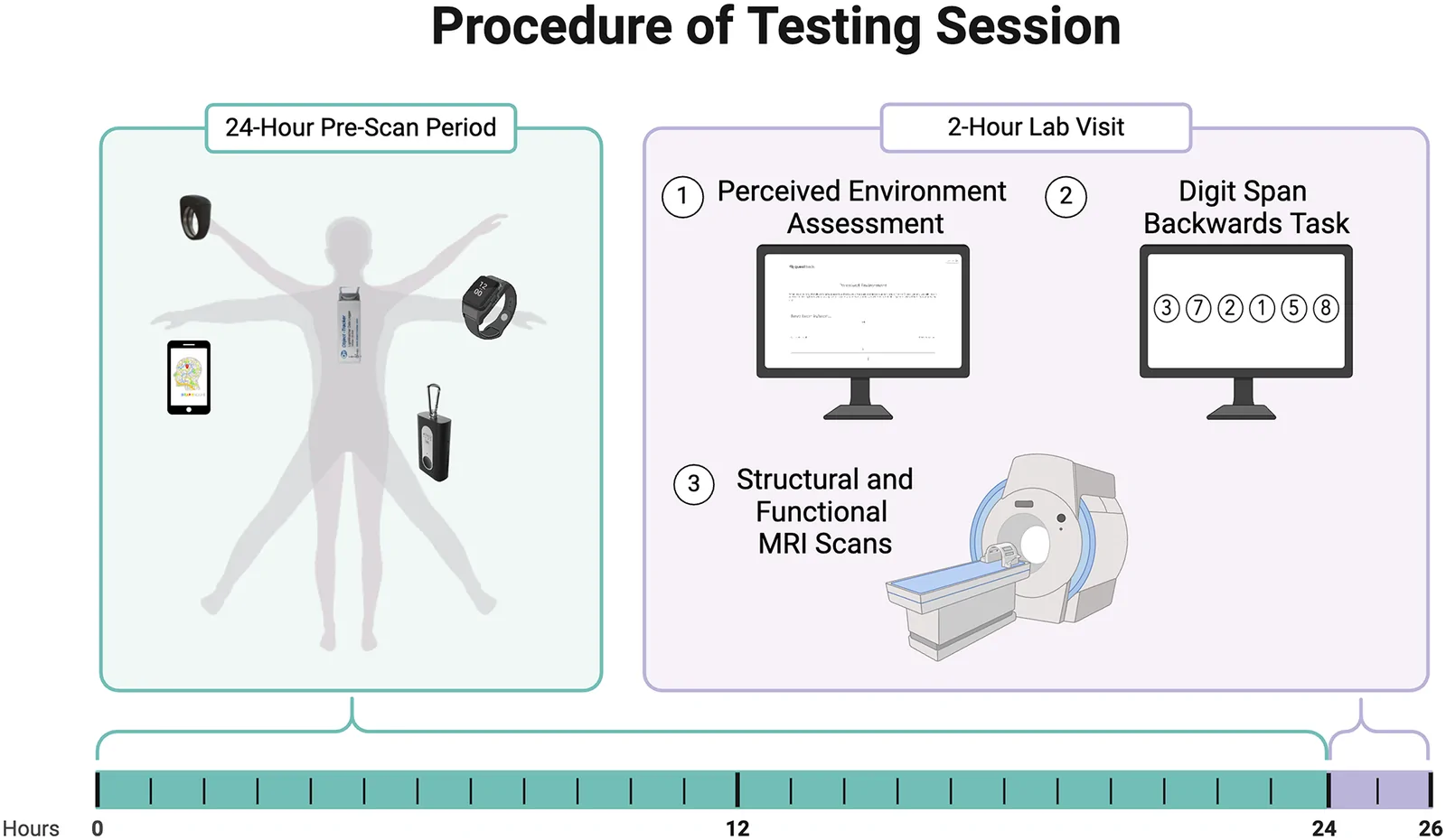
Participants complete 25 testing sessions each including a pre-scan period (24 h) and a lab visit (2 h). Created in BioRender. Falkenstein, K. (2025) https://BioRender.com/n27z672

Prescreened participants visit the lab before the first testing session to sign consent forms, and to receive instructions on the use of the devices and applications utilized in this study. Over three to six months, each participant completes 25 testing sessions (~ 2 sessions per week, depending on availability of the participant and the MRI scanner), including a 24-hour pre-scan period and a lab visit with both structural and functional MRI scans (see Fig. 1). For the pre-scan period, participants are instructed to activate all devices and applications at least 24 h before each lab visit to collect environmental, physiological, and behavioral data during the 24 h preceding MRI acquisition. The set of devices and applications include GPS tracking on a provided study phone, a smart watch to monitor relevant health-related parameters and sleep, devices for assessing indoor and outdoor air quality, a smart ring to track electrodermal activity, a light tracker to measure a number of light parameters, and an app for geographic ecological momentary assessment (GEMA) including cognition and affect. If consent is provided, we additionally collect GPS data throughout the duration of the study by installing a custom-made app (see BrainScape app in Table 1) on the participants’ personal phones. The functions of the devices and apps are described in more detail in Table 1 and the following chapters (Environmental data, Behavioral data, Physiological data). During the pre-scan period, participants receive notifications on the provided study phone to complete short GEMA questionnaires approximately every two hours. These questionnaires include a 2-minute working memory updating task, questions about stress, affect, and rumination, and prompts to report their subjective perceptions of their current environment. Each lab visit following the pre-scan period includes a questionnaire about the perceived environment and emotions, a memory task, and MRI scanning. Participants receive the set of devices for the full duration of the study.

**Table 1:** Descriptions of devices and applications used to acquire environmental, physiological, and psychosocial data during the pre-scan period

Device/Application	Measures and usage
**iPhone 7**,** Apple Watch Series 5**,** and Health app** *(Apple Inc.*,* Cupertino*,* California*,* United States)*	The Apple Watch tracks various health-related metrics, such as heart rate, physical activity, sleep, and ambient noise levels in the participant’s environment. It automatically transfers and stores the data in the Health app on the connected iPhone. All relevant apps listed below are installed on the study-provided iPhone.
**air-Q pro and air-Q manager app** *(Corant GmbH*,* Leipzig*,* Germany)*	The air-Q device monitors gases, particles, and chemicals in the air of participants’ homes. Participants set up the device in the room where they spend most of their time at home. Using the air-Q manager app on the provided smartphone, they connect the device to their Wi-Fi to access the measured data both in the app and through the air-Q cloud service. Additionally, all measured data is stored on an SD card inserted inside the device. Participants are instructed to turn off the device’s LED indicators and refrain from frequently checking the app to avoid any behavioral changes in response to the air quality data. It is recommended that participants keep the air-Q device powered on throughout the entire study period, as setting up the device with Wi-Fi is time-consuming and continuous operation prevents complications with the measurement of air quality.
**Atmotube PRO and Atmotube app** *(Atmotech Inc.*,* San Francisco*,* California*,* United States)*	The Atmotube PRO is a portable air quality monitor that detects fine particles and volatile organic compounds (VOCs) both indoors and outdoors. Once turned on, the device automatically connects to the Atmotube app installed on participants’ study phones. When connected, the data is synchronized and stored on the phone. At the start of each pre-scan period, participants are instructed to turn on the Atmotube, open the app, verify that the Atmotube is connected in order to ensure accurate data collection and carry the device with them during the pre-scan period.
**Nuanic ring and Nuanic app** *(Nuanic Oy*,* Tampere*,* Finland)*	The Nuanic ring is a bio-sensor measuring electrodermal activity (EDA). The ring transfers the EDA data from its memory to the Nuanic app installed on the participants’ study phones via Bluetooth. The data is stored on the study phone.
**Light-Watcher** *(Wolf Technologieberatung*,* Perchtoldsdorf*,* Austria)*	The Light-Watcher records light irradiance in 5 spectral bands (ultraviolet, red, green, blue, infrared), acceleration in 3 axis (actimeter) and temperature. The data is stored on the device.
**BrainScape app** *(Max Planck Institute for Human Development*,* Berlin*,* Germany)*	BrainScape is a custom-made app that integrates data from different data sources including continuous GPS tracking and measurement of noise and air quality (retrieved from the Atmotube). Furthermore, the app prompts participants seven to eight times within each pre-scan period to answer questions about stress, mood, and rumination, report their subjective perceptions of their current environment and to complete a 2-minute working memory updating task. The app is installed on the provided study phone.
**Workout app** *(Apple Inc.)*	The Workout app installed on the Apple Watch and the iPhone tracks individual workout sessions. Participants are required to start a new recording at the beginning of a workout session (e.g. running, swimming, Pilates etc.). The data is automatically synced with the Health app.
**Pillow app** *(Neybox Digital Ltd.*,* Limassol*,* Cyprus)*	The Pillow app is installed on the Apple Watch and monitors sleep duration, quality, and stages. The data is automatically transferred and stored in the Pillow app on participants’ study phones.
**GPS-Tracker Pro app** *(Apple App Store*,* developed by Marvin Wagner)*	The GPS-Tracker Pro is an app installed on the study phone that tracks participants’ locations during the pre-scan periods. Participants are required to start the recording at the beginning of each new pre-scan period. The data is stored locally in the app on the phone.

To ensure the integrity of data from the pre-scan period, there must be at least 26 h between lab visits. Participants must maintain high compliance in wearing sensors, completing GEMAs, and adhering to the study’s schedule. Despite frequent data checks and exports, occasional data loss due to technical challenges remains possible. To mitigate participant fatigue, we make every effort to schedule MRI appointments according to each participant’s availability, ensuring flexibility throughout the study. At the end of the study, participants receive 1100€: 600€ for the 25 lab visits, 400€ for wearing the devices during the pre-scan period, and a 100€ bonus for completing all 25 testing sessions. Additionally, participants who consent to GPS data collection by installing the BrainScape app on their personal phone receive an extra 50€.

In the following sections, we provide a detailed description of the various types of data being collected, along with the devices and apps used for data acquisition. Additionally, a complete list of all variables and data formats is available here https://day2day-environment-project.mpib.berlin/.

### Brain data

MRI data are acquired on a 3 Tesla Siemens Magnetom Tim Trio MRI scanner (Erlangen, Germany) using a 32-channel head coil at the Max Planck Dahlem Campus of Cognition (MPDCC) of the Max Planck Institute for Human Development in Berlin, Germany.

#### T1 sequence

T1-weighted structural images are acquired using a three-dimensional T1-weighted magnetization prepared gradient-echo sequence (MP-RAGE) with the following parameters: Repetition time (TR) = 2500 ms; echo time (TE) = 4.77 ms; inversion time (TI) = 1100 ms; acquisition matrix = 192 (R > > L) x 256 (A > > P) x 256 (F > > H) mm^3^; flip angle (FA) = 7°; 1 × 1 × 1 mm^3^ voxel size. The sequence duration is 9:20 min. Participants are instructed to keep their eyes closed.

#### Functional resting state sequence

Resting-state functional magnetic resonance imaging involves acquisition of 150 T2*-weighted echo-planar images (EPI) sensitive to BOLD contrast using the following parameters: TR = 2000 ms; TE = 30 ms; acquisition matrix = 216 × 216 × 129 mm^3^; FA = 80°; slice thickness = 3.0 mm; distance factor = 20%, field-of-view (FOV) = 216 mm; 3 × 3 × 3 mm^3^ voxel size; 36 axial slices, using generalized auto-calibration partially parallel acquisition algorithm (GRAPPA) factor of 2. The sequence duration is 5:08 min. Participants are instructed to keep their eyes closed.

#### Functional task-based sequences

Functional task-based sequences are acquired using the same parameters as the resting-state sequence.

**Fearful Faces Task (FFT):** This task involves the acquisition of up to 250 T2*-weighted EPIs. The total sequence duration is 8:28 min but it is stopped once the task is completed. Because acquisition is terminated once the task is completed, the number of volumes acquired may vary slightly below this nominal maximum across sessions.

We employ a modified version of the Fearful Faces Task (FFT) to assess amygdala responses to fearful and neutral facial expressions [41, 42]. Participants are presented a series of 15 male and 15 female faces (obtained from the FACES database; [43]), each displaying either a fearful or neutral expression. The paradigm comprises 20 blocks randomly distributed across one of four conditions: fearful, neutral, masked fearful, masked neutral. For the fearful and neutral conditions, 6 face stimuli are presented for 1 s followed by a fixation cross for 300 ms. For the masked fearful and masked neutral conditions, each trial begins with a 50 ms fearful or neutral face, respectively, followed by a 966 ms neutral face, with a total of 6 trials per block. After each block, a fixation cross is displayed for 9s. To monitor participants’ attention, the fixation cross turns red on two occasions, and participants are instructed to press a button on the response box whenever they see the red cross. The order of stimuli is randomized across 10 different versions of the FFT.

**Perceptual Speed Task:** This task involves the acquisition of up to 300 T2*-weighted EPIs. The total sequence duration is 10:08 min but is stopped once the task is completed. Because acquisition is terminated once the task is completed, the number of volumes acquired may vary slightly below this nominal maximum across sessions.

In the Perceptual Speed Task [44–46], participants are asked to determine whether briefly displayed numbers are odd or even and respond via button box. The odd numbers used in the task are “3”, “5”, “7” and “9” while the even numbers are “2”, “4”, “6” and “8”. Each session consists of 8 blocks, each containing 16 trials (every number is presented twice per block). Trials begin with a fixation cross for a jittered duration of either 500 ms, 1 s, 1.5 s, or 2 s, equally distributed throughout each block. Each number is displayed for 66 ms in a layout mimicking the seven-segment “8” found on calculators. After each number is shown, a mask appears for 2 s that consists of this “calculator 8” symbol overlaid with lines extending in all 10 possible directions to obscure the number. Participants can indicate their odd or even responses during the masking period.

Both task sequences are presented via a projector-mirror system, with responses collected through a response box. They are implemented using the software Presentation (version 19.0).

**Fig. 2:**
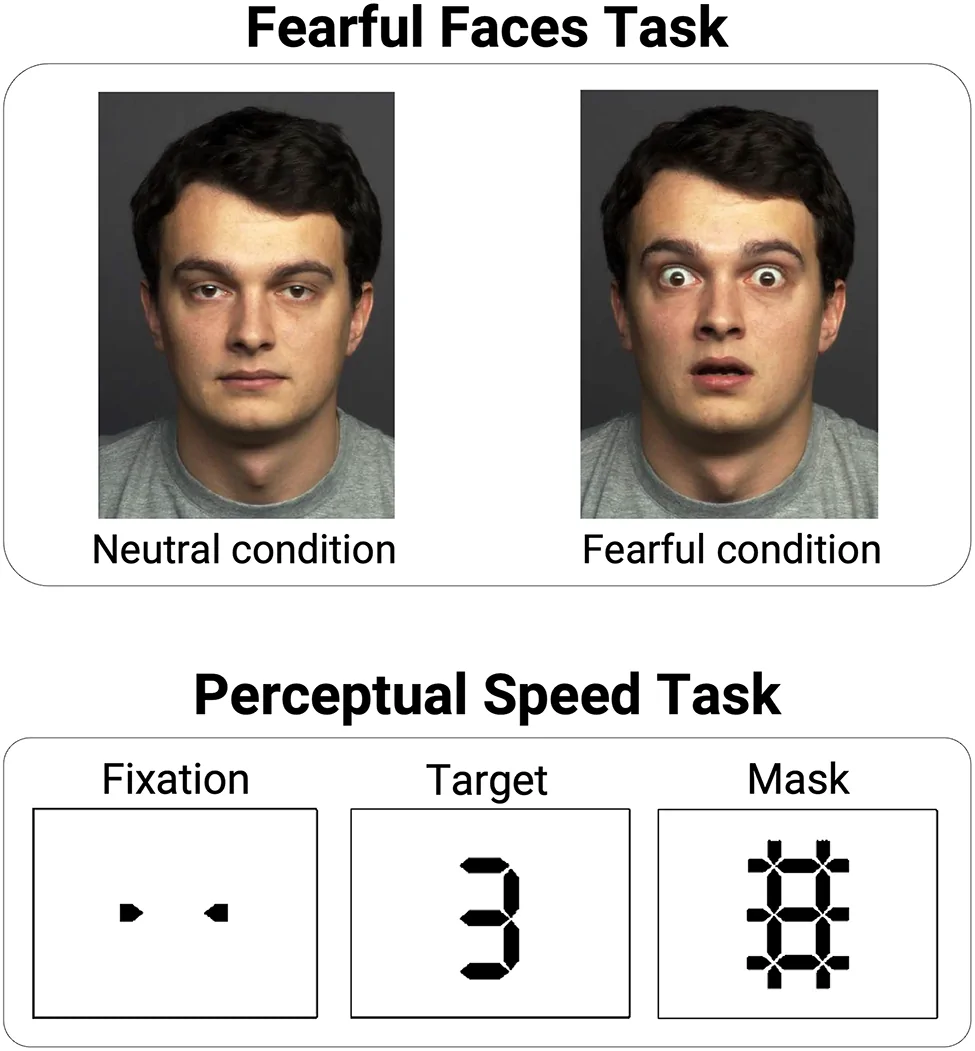
Example stimuli from the Fearful Faces Task (top) and the Perceptual Speed Task (bottom)

#### Quantitative multi-parameter mapping

In order to identify brain regions where micro-structural changes might be associated with alterations in environmental parameters, we additionally acquire quantitative multi-parameter mapping (MPM). The MPM scan with 1.3 mm isotropic resolution includes three different 3D multi-echo fast low-angle shot (FLASH) gradient-echo acquisitions [47], including magnetization transfer saturation (MT), proton density (PD), longitudinal relaxation rate (R1 = 1/T1), and transverse relaxation rate (R2* = 1/T2*) with a FOV of 224 × 256 mm^2^. In order to minimize acquisition time, parallel imaging is used in the phase-encoding direction (anterior-posterior) with a GRAPPA factor of 2, combined with a 6/8 partial Fourier acquisition in the partition direction (left-right). The readout bandwidth is 470 Hz/pixel, allowing for six echoes between 2.46 and 14.78 ms for all three acquisitions. The contrast parameters are as follows: TR = 37 ms and FA = 6° for MT-weighted images with a Gaussian off-resonance RF pulse (500°, 10 ms, 1,200 Hz off-resonance, 192 Hz bandwidth) prior to non-selective excitation; TR = 18 ms and FA = 4° for PD-weighted images; and TR = 18 ms and FA = 25° for T1-weighted images. To enable bias field correction, an RF transmit (B1+) map is acquired for all runs, with a FOV exactly matching the MPM scans at a resolution of 4 × 4 × 5 mm^3^. The B1 + map is derived from spin-echo/stimulated echo acquisitions using a standard vendor sequence [48]. The total sequence duration is 12:36 min. Participants are instructed to keep their eyes closed.

#### Hippocampus high-resolution sequence

To quantify hippocampal subfield volumes, a high-resolution PD sequence is used with the following parameters: TR = 6500 ms; TE = 16 ms; acquisition matrix = 165 × 60 × 206 mm^3^; 0.4 × 0.4 mm in-plane resolution; 2 mm slice thickness; 30 slices. The sequence duration is 4:15 min. Participants are instructed to keep their eyes closed.

#### Diffusion tensor imaging (DTI)

Two transverse DTI sequences with opposite polarity of the phase encoding (PE) direction (i.e., AP and PA) are acquired using a single-shot diffusion-weighted spin-echo-refocused echo-planar imaging sequence. The AP sequence includes 10 non-diffusion weighted images (b = 0s/mm^2^), 30 diffusion-weighted images with b = 710s/mm^2^, and 60 diffusion-weighted images with b = 2850s/mm^2^ (total duration: 16:41 min). The PA sequence consists of 6 diffusion-weighted images with b = 1000s/mm^2^ (total duration: 1:29 min). The following parameters are consistent across PE directions: TR = 9700 ms; TE = 120 ms; FOV = 224 × 224 × 124 mm^3^, voxel size = 2 × 2 × 2 mm^3^; 62 slices using GRAPPA factor 2 [49]. Participants are instructed to keep their eyes closed.

### Behavioral data

Participants receive seven to eight prompts on random occasions during the pre-scan period from the BrainScape app installed on the study-provided smartphone. These prompts involve momentary assessments, including queries about stress, mood, rumination, subjective perceptions of their current environment, and a working memory task (Spatial Updating Task).

At the beginning of each lab visit, participants are required to complete an online questionnaire (hereon referred to as the pre-MRI questionnaire). This questionnaire collects data on their perceptions of the environment over the preceding 24 h, as well as their emotional and mental state, and is followed by the Digit Span Backwards Task.

#### Perceived environment

As part of the **BrainScape questionnaire**, participants rate on a continuous visual analogue scale from 0 to 100 (not at all — very much), how satisfied they are with the visible environment, the air quality, and the soundscape that surrounds them. Additional questions address details of their immediate environment, such as whether they are currently indoors or outdoors, recent time spent outside, and the activities they have just engaged in.

In the **pre-MRI questionnaire**, participants rate their exposure to various environmental elements over the past 24 h on a continuous visual analogue scale from 0 to 100 (less than usual — more than usual) to indicate the frequency and intensity of their exposure to natural features (e.g., parks, forests, water) and environmental stressors (e.g., loudness, air pollution, traffic). Additionally, participants can indicate if they were not exposed to or annoyed by a particular element.

#### Cognitive performance

The **Digit Span Backwards Task** is completed on a computer during the lab-based session. The task is used to assess working memory capacity. Participants are presented with digit sequences and have to recall them in the reversed order by selecting the digits from a circle of digits with the mouse. Participants must complete a minimum of 2 and a maximum of 8 practice trials. The practice session ends and progresses to the test session once a correct response is given. The minimum length of digits is two (e.g. 93) and the maximum length is 14 (e.g. 31587296926725). Each test block contains two trials. If the participant correctly answers one trial in a block, the number of digits increases for the next block. If the participant cannot answer any trials correctly for a certain digit length, the task ends [50, 51]. Due to an error in the task script, the maximum length was limited to 8 digits for data collected between January 29, 2024, and November 12, 2024.

The **Spatial Updating Task** is presented at each prompt within the scope of the GEMA assessment. The four digits (0–9) are displayed simultaneously in a 2 × 2 grid for 4000 ms. After a 500 ms interval, a sequence of five updating operations (additions or subtractions from − 8 to + 8) are presented in the corresponding cells (presentation times 2000 ms; interstimulus interval 500 ms). Participants are required to apply these operations to the corresponding digits, updating and memorizing the results. Each operation targets a different cell than the previous one, ensuring no cell is updated twice in a sequence. The intermediate and final results all fall within the range of zero to nine. At the end of each trial, participants enter the final results into the grid cells and receive performance feedback. The task includes two trials [45, 52, 53].

#### Self-reported emotions/psychological state

At the beginning of the study, participants complete the **Connectedness to Nature Scale** (CNS; [54]) once and are asked to list three words they associate with “nature” and “urban”. Further questions assess the amount of time participants spend in natural environments and in traffic, the activities they typically engage in while in nature, and their common modes of transportation.

Furthermore, we use an abridged version of the **Emotional Recall Task** to assess the participants’ recalled emotional experiences [55]. Participants first recall five words that describe their feelings over the past 24 h. They then indicate, on a continuous visual analogue scale from 0 to 100 (0 = not at all, 100 = completely), how frequently they experienced each feeling during that time. Finally, participants rate each of the five feelings on two scales: calm — excited and unpleasant — pleasant, each ranging from 1 to 9.

As an additional measure to assess participants’ self-reported emotions, we use a questionnaire developed by Stemmler et al. [56]. Participants rate the intensity of their emotions on six unipolar scales (ranging from 0 = “not applicable” to 10 = “completely applicable”) and five bipolar scales (ranging from − 5 to 5). The scales measure a variety of emotional experiences, including feelings of shame, fear, sadness, happiness, and anger, as well as bodily sensations like a pounding heart. Additionally, they capture feelings of arousal and valence (e.g., tense versus relaxed, active versus tired, positive versus negative), cognitive states (e.g., alert versus confused), and motivational states (e.g., interested versus bored).

The Emotional Recall Task and Stemmler questionnaire are always completed during the lab appointment, as part of the pre-MRI questionnaire, before MRI acquisition.

As part of the **BrainScape questionnaire**, positive and negative affect is measured using 14 items (i.e., happy, relaxed, interested, satisfied, rested, balanced, stimulated, floppy, sad, worried, frustrated, nervous, twitchy, and angry). Each item is rated on a continuous visual analogue scale (VAS) from 0 to 100 (0 = very slightly or not at all, 100 = extremely). Affect items were selected from validated item lists [57, 58], cover both high-arousal and low-arousal negative affect [59], have been widely used in research with older participants [60], and are known to capture hour-to-hour fluctuations [61, 62]. In addition to affect, we assess loneliness with four items (e.g., “I feel lonely”, “I feel isolated from others”) which are also rated on a VAS from 0 to 100 (0 = don’t agree at all, 100 = agree completely). These items were adapted from the UCLA Loneliness Scale [63], and have also been validated in a number of ambulatory assessment studies [64].

We measure rumination, defined as the recurrence of unwanted or negative thoughts, using three items adapted from previous studies [65–68] and the Stress Coping Inventory [69]. Participants rate how much each statement — “I could not get certain thoughts out of my mind,” “I kept thinking about something over and over again,” and “I had difficulties suppressing thoughts about myself” — reflects their recent thoughts and feelings. Additionally, momentary paranoia is assessed with the Brief State Paranoia Checklist, which consists of three items (“I have to be careful around others”, “People try to unsettle me”, “Strangers and friends look at me critically”) to capture changes in paranoia levels [70].

Further questions cover subjective age, indicating how old a person feels [71, 72], perceived control, which reflects beliefs about one’s ability to shape and impact life circumstances [60], and self-rated health.

#### Digital media usage

Participants are instructed to take pictures of their screen time on various devices, such as phones, computers, tablets, consoles, and TVs, capturing the minutes of usage for each device type during the pre-scan period.

### Physiological data

#### Electrodermal activity

Measurable changes in skin conductance, referred to as Electrodermal Activity (EDA), indicate the activity of the sympathetic branch of the autonomic nervous system, which is associated with emotional responses, cognitive functions, and affect [73]. EDA is also a recognized measure of an individual’s stress level [74, 75]. In this study, we collect EDA data during the pre-scan period using the Nuanic ring or its predecessor, the Moodmetric ring.

#### Fluid intake

To assess fluid intake, participants report in the BrainScape questionnaire their intake of water, soft drinks (such as soda, cola, and juice), stimulant drinks (including coffee, energy drinks, tea, and cocoa), and alcoholic beverages.

#### Heart rate

We use the Apple Watch to measure several physiological parameters related to cardiovascular health, including heart rate, heart rate variability, and resting heart rate. Heart rate is the continuous measure of the number of beats per minute, reflecting overall cardiovascular activity. Heart rate variability measures the variation in time between consecutive heartbeats, which is an indicator of autonomic nervous system function and overall heart health. Resting heart rate represents the number of heartbeats per minute when a person is at rest, providing insights into baseline cardiovascular fitness and stress levels.

#### Physical activity

The Apple Watch tracks the number of steps taken throughout the day. Additionally, the device records detailed information on workout activities, including the type, duration, and intensity of exercises performed.

The device also records two types of energy burned: Resting (basal) energy burned, which represents the amount of energy expended while at rest, and active energy burned, which accounts for the energy used during physical activities.

#### Sleep

Using the Pillow app, installed on the Apple Watch and the study phone, we collect data on participants’ sleep behavior. The app estimates and records various sleep metrics, including total sleep duration and sleep stages (light, deep, and REM sleep) throughout the night. In addition, as part of the first BrainScape questionnaire, each day participants answer questions about their perceived sleep experience, such as the time they went to bed, how long it took to fall asleep, when they woke up, whether they slept through the night, and their subjective rating of sleep quality.

#### Menstrual cycle

Menstrual cycle data are collected through the Health app installed on the study phone. Female participants manually enter information about the start and end date of their period.

### Environmental data

Several environmental measures are collected continuously throughout the pre-scan period using the devices and applications referenced in Table 1; Fig. 2.

#### Air quality

We continuously collect air quality data in participants’ homes throughout the study using the air-Q device. In addition, participants monitor the air quality in their immediate surroundings (indoors and outdoors) by carrying a portable air quality monitor (Atmotube) with them during the pre-scan period.

#### GPS-based location

We collect participants’ location data during the pre-scan period using the GPS-Tracker Pro and the BrainScape app installed on the study-provided phone. With participants’ consent, continuous GPS data is also collected throughout the study via the BrainScape app installed on their personal phones. To guarantee accurate data collection, participants must ensure that the BrainScape app remains active in the background.

#### Environmental audio exposure

Using the Apple Watch, we continuously assess the ambient noise levels in decibels (dB) in the participants’ environment during the pre-scan period.

#### Light exposure

In order to measure the participants’ exposure to light during the pre-scan period, we use the Light Watcher to record light irradiance in five spectral bands: ultraviolet (UV), red, green, blue, and infrared (IR). In addition, the Light Watcher also measures acceleration in three axes (actimeter) and air temperature.

### Sociodemographic data

In addition to standard demographic questions, including on education and income, we collect information about participants’ current environmental living conditions as well as living conditions during their upbringing based on the Urbanicity Score by Lederbogen et al. [8]. We also ask participants about their use of prescribed medications, stimulant drugs or substances, and alcohol consumption.

## Utility and discussion

The Day2Day Environment project aims to provide unique insights into how the complex interplay between multiple environmental exposures influences the brain and well-being, while also identifying confounding variables that may affect the reliability of MRI measurements. To achieve this, we are gathering a longitudinal, multimodal dataset that combines neuroimaging with physiological, behavioral, cognitive, affective, and environmental data across 25 timepoints per participant, capturing variability over time. Given that real-world environments expose individuals to multiple “active ingredients” [1] simultaneously, the richness of this dataset allows researchers to analyze both singular and combined exposures in relation to brain outcomes and mental health.

To uncover these complex relationships, we plan to use machine learning methods such as feature selection to identify the most relevant environmental predictors and to explore possible interactions between them. Because of the modest number of participants, we treat machine-learning-identified predictors as exploratory/hypothesis-generating rather than confirmatory. Given the high dimensionality of, and potential multicollinearity among, the environmental, behavioral, and physiological predictors, appropriate feature-selection and dimensionality-reduction approaches will be applied. Additionally, we will employ linear mixed-effect models to directly assess how various environmental factors relate to brain structure and function, while accounting for the within-subject design. The linear mixed-effects models derive their statistical power primarily from within-person contrasts across approximately 750 total testing sessions. Multiple-testing correction, cross-validation procedures suited to the repeated-measures structure, and missing-data handling will be addressed accordingly. As this protocol paper describes the study design and available measures rather than a statistical plan, the specific outcomes and pre-registered statistical models will instead be defined separately for each hypothesis-driven or exploratory analysis based on this dataset.

Several physiological and seasonal factors could act as confounders of MRI-based brain measures. Evidence for such effects is mixed: studies have reported short-term effects of exercise [20], (de)hydration [21, 22], caffeine intake [23, 24], or the menstrual cycle [25] on the brain. However, in earlier studies using a very similar design to ours, Karch et al. [29] found that only time since the first scan and time of day were reliable predictors of within-person variance in brain volume; caffeine intake, fluid intake, and estradiol did not emerge as significant predictors, and testosterone showed only weak predictive evidence. Similarly, Di et al. [32] reported no detectable influence of weather on brain function beyond effects on the MRI scanner’s baseline signal. As the present study collects data on these possible confounders, we will be able to test the relevance of these factors directly and include them as covariates in our models.

One limitation of prior research has been the reliance on static assessments of environmental exposure [76], overlooking the fact that individuals experience a variety of different environments in a given day. This is particularly relevant for exposures with high spatial and temporal variation, like air and noise pollution. Using GEMA alongside environmental and physiological sensors, as well as tracking movement patterns, allows for real-world, real-time data collection and the extensive use of additional map-based information when employing a Geographic Information Systems (GIS) approach, which enhances the ecological validity of the study.

There is a large body of literature focusing on brain dynamics for very short timescales (seconds to minutes, e.g. learning studies) and long timescales (years to decades, e.g. developmental and aging studies), yet we know far less about brain variability across days and months. Accordingly, we focus on environmental, behavioral, and physiological monitoring in the 24 h immediately preceding MRI acquisition. This window targets proximal influences on brain measures. The repeated MRI scans within individuals over time provide the opportunity to examine within-person variability and potentially, brain plasticity across daily, weekly, and monthly periods in response to varying daily activities and environments. Because each participant contributes 25 repeated sessions, we expect atypical pre-scan periods to primarily inflate variance rather than introduce systematic bias.

This dataset also has the potential to become a valuable resource for the broader scientific community, enabling researchers from diverse fields to explore a wide range of research questions, as demonstrated by the utility of the previous Day2Day dataset [28–38]. With its unique combination of longitudinal human-environment interaction data, the dataset is expected to support inquiries not only within environmental neuroscience but also in disciplines like public health and urban planning.

Beyond advancing scientific understanding, the findings from the data can be especially valuable for developing practical interventions in everyday life. For instance, urban planners and policymakers could use these findings to create environments that actively support mental and brain health. Additionally, results could inform public health strategies to encourage behaviors that positively influence brain structure and function.

### Challenges and limitations

This study’s demanding schedule and reliance on high levels of participant commitment over 25 testing sessions, including the pre-scan periods, may lead to participant fatigue and selective dropouts. Additionally, insufficiently charged devices that switch off during the 24-hour pre-scan period and require recharging can result in temporary data gaps. To mitigate these risks, we provide an extensive briefing during recruitment to set clear expectations about what participation would involve and a completion bonus of €100. Additionally, we monitor data quality and device compliance throughout the study via regular checks at each lab appointment, which allows us to identify declining engagement and re-engage participants where possible. Nonetheless, some dropout and missingness are expected over the course of the study and will be documented and reported transparently in subsequent publications using this dataset. To date, only 6% of participants have dropped out before completing all 25 sessions.

The geographic limitation of all participants residing in or close to Berlin, may restrict the range of environmental contexts represented in the sample and therefore the generalizability of the findings.

The study’s within-person design and the large number of testing sessions per participant strengthen reliability, however, the relatively small sample size (30 participants) may limit generalizability. However, it should be noted that previous studies focusing on dense within-subject sampling have considerably lower samples sizes (*n* = 8, [77]; *n* = 4, [78]; *n* = 6, [28]; *n* = 10, [80]; *n* = 1, [79]).

## Conclusions

The Day2Day Environment project offers a novel approach to studying the impact of real-world environmental factors on brain structure, function, and mental well-being, including cognition and affect. By capturing a comprehensive range of physiological, behavioral, cognitive, affective, and environmental data alongside various brain measures across multiple time points, this dataset enables us to address critical gaps in understanding how dynamic, everyday environments shape the brain. With the ability to examine both singular and combined exposures, we aim to reveal how the brain responds to daily variations in air quality, noise, light, and other factors. These findings have broad implications not only for environmental neuroscience but also for designing interventions and public policies that promote mental health and brain resilience in diverse environments.

## Data Availability

While European legislation restricts us from storing the data in public repositories, we welcome collaboration with the international research community. Data can be shared upon reasonable request for formal collaborations, provided that appropriate data transfer agreements are in place in compliance with German and European law. For collaboration inquiries and data requests, please contact Prof. Simone Kühn (kuehn@mpib-berlin.mpg.de)The dataset is organized in accordance with the Brain Imaging Data Structure (BIDS). Please find a complete list of all variables and data formats under the following link: https://day2day-environment-project.mpib.berlin/.
